# Asymmetric effects of native and exotic invasive shrubs on ecology of the West Nile virus vector *Culex pipiens* (Diptera: Culicidae)

**DOI:** 10.1186/s13071-015-0941-z

**Published:** 2015-06-16

**Authors:** Allison M. Gardner, Brian F. Allan, Lauren A. Frisbie, Ephantus J. Muturi

**Affiliations:** Department of Entomology, University of Illinois at Urbana-Champaign, 505 S. Goodwin Ave., Urbana, IL 61801 USA; School of Integrative Biology, University of Illinois at Urbana-Champaign, 505 S. Goodwin Ave., Urbana, IL 61801 USA; Illinois Natural History Survey, 1816 S. Oak St., Champaign, IL 61820 USA

**Keywords:** *Culex pipiens*, Invasive plants, Habitat selection, Ecological trap, Mosquito ecology

## Abstract

**Background:**

Exotic invasive plants alter the structure and function of native ecosystems and may influence the distribution and abundance of arthropod disease vectors by modifying habitat quality. This study investigated how invasive plants alter the ecology of *Culex pipiens*, an important vector of West Nile virus (WNV) in northeastern and midwestern regions of the United States.

**Methods:**

Field and laboratory experiments were conducted to test the hypothesis that three native leaf species (*Rubus allegheniensis*, blackberry; *Sambucus canadensis*, elderberry; and *Amelanchier laevis*, serviceberry), and three exotic invasive leaf species (*Lonicera maackii*, Amur honeysuckle; *Elaeagnus umbellata*, autumn olive; and *Rosa multiflora*, multiflora rose) alter *Cx. pipiens* oviposition site selection, emergence rates, development time, and adult body size. The relative abundance of seven bacterial phyla in infusions of the six leaf species also was determined using quantitative real-time polymerase chain reaction to test the hypothesis that variation in emergence, development, and oviposition site selection is correlated to differences in the diversity and abundance of bacteria associated with different leaf species, important determinants of nutrient quality and availability for mosquito larvae.

**Results:**

Leaf detritus from invasive honeysuckle and autumn olive yielded significantly higher adult emergence rates compared to detritus from the remaining leaf species and honeysuckle alleviated the negative effects of intraspecific competition on adult emergence. Conversely, leaves of native blackberry acted as an ecological trap, generating high oviposition but low emergence rates. Variation in bacterial flora associated with different leaf species may explain this asymmetrical production of mosquitoes: emergence rates and oviposition rates were positively correlated to bacterial abundance and diversity, respectively.

**Conclusions:**

We conclude that the displacement of native understory plant species by certain invasive shrubs may increase production of *Cx. pipiens* with potential negative repercussions for human and wildlife health. These findings may be relevant to mosquito control and invasive plant management practices in the geographic range of *Cx. pipiens*. Further, our discovery of a previously unknown ecological trap for an important vector of WNV has the potential to lead to novel alternatives to conventional insecticides in mosquito control by exploiting the apparent “attract-kill” properties of this native plant species.

## Background

Exotic shrubs, aided mostly by human activities, have become dominant features of urban and suburban landscapes in North America [1, 2]. Deliberate introduction of non-native turf grass lawns and ornamental plants remains a common practice in residential neighborhoods despite the well-documented potential for these species to become invasive in their introduced ranges [3]. On a broader scale, the disturbed structure of urban communities has given rise to an altered natural selection process that often functions to the competitive detriment of native species [4]. As a consequence, propagation of exotic invasive plants (hereafter “invasive plants”) may reduce the ecosystem services typically provided by native plant communities, including nutrient cycling, prevention of stream erosion, air filtration, and preservation of wildlife diversity [5–9].

There is also recent evidence that invasive plants can cause ecological cascades that alter human risk of exposure to diseases vectored by arthropods. For example, invasive plants may enhance the risk of exposure to tick-borne pathogens by increasing the abundance and encounter frequency of ticks and their vertebrate hosts [10–12]. In mosquito-borne disease systems, changes in habitat complexity resulting from plant invasions may alter oviposition site selection by some vector species [13], and inputs of fruits and leaves of some terrestrial invasive plant species into aquatic larval habitats may provide a high-quality nutritional base for mosquito larvae [14]. Yet despite the strong potential for vectors to interact with invasive plants, we have limited understanding of the diversity of potential mechanisms by which these plants alter vector ecology and the consequences for human health.

Container-dwelling mosquitoes provide an ideal model system for understanding the effects of native and invasive plants on mosquito ecology and the risk of human exposure to vector-borne pathogens. *Culex pipiens pipiens* Linnaeus (Diptera: Culicidae), an important vector for West Nile virus in urban landscapes throughout the northeastern and midwestern United States [15], oviposits in a variety of natural and artificial containers such as small ponds, discarded tires, and storm water catch basins [16, 17]. These habitats are mainly fueled by plant-based detritus from the surrounding terrestrial vegetation. This often includes leaves of non-native understory plant species (Gardner, pers. obs.), which commonly are introduced to residential environments for home landscaping and often become invasive in small urban forest fragments [18]. Detritus type and quantity determine the composition and abundance of microbial communities that form in container habitats as microbes break down terrestrial leaf litter [19–23]. In turn, these bacteria and fungi provide a direct food source for mosquito larvae [24, 25] and influence oviposition behavior of gravid female mosquitoes through emission of oviposition attractants and stimulants [26, 27]. Thus, detritus from terrestrial plants and its associated microbes play a critical role in determining vector distribution, relative abundance, and life history traits that are important for vector-borne pathogen transmission including adult body size, longevity, biting rates, and vector competence [28–31].

It has been proposed that detritus from invasive plants benefits mosquito vectors and enhances vector-borne disease transmission potential because these leaves decompose faster and facilitate more rapid microbial growth compared to native plants [32–34]. In this study, we test the hypotheses that A) leaf detritus of three native and three invasive shrubs asymmetrically affects the emergence rates, development, and oviposition site selection by *Cx.pipiens*, and B) variation in emergence, development, and oviposition site selection is correlated with differences in the diversity and abundance of bacterial flora among leaf detritus types.

## Methods

### Selection of plant species

Six focal plant species were selected among shrubs common within the geographic range of *Cx. pipiens* [35]. The invasive shrubs were *Lonicera maackii* (Dipsacales: Caprifoliaceae; Amur honeysuckle), *Elaeagnus umbellata* (Rosales: Elaeagnaceae; autumn olive), and *Rosa multiflora* (Rosales: Rosaceae; multiflora rose). Found in both natural and domestic environments – including forest fragments, landscaped parks, and residential neighborhoods – these three exotics are highly invasive throughout much of the United States, and in some areas of the northeast and Midwest dominate over 80 % of land cover [36, 37]. The native shrubs were *Rubus allegheniensis* (Rosales: Rosaceae; blackberry), *Sambucus canadensis* (Dipsacales: Adoxaceae; elderberry), and *Amelanchier laevis* (Rosales: Rosaceae; serviceberry), all three of which are common understory species in mixed deciduous forests and residential landscapes.

### Attractiveness of leaf species for*Cx. pipiens* oviposition

Six oviposition traps [38] each containing 4 L of tap water and 80 g of fresh whole leaves of one of the six shrub species were placed 1 m apart from each other in partial shade at five sites located within a 5 km radius in residential Champaign-Urbana, Illinois. The 30 oviposition traps were monitored for egg rafts daily and the number of egg rafts collected in each substrate from June 24 to August 5, 2013 (39 days) was recorded. This design, which was informed by a smaller pilot study performed in 2012, tested for temporal variation in the attractiveness of the leaf species to gravid females based on the number of egg rafts collected, which may vary as the microbial and chemical composition of the infusion changes with decomposition of the leaves [32, 33].

Data analyses were conducted in SAS 9.3 (SAS Institute Inc., Cary, NC). A general linear mixed model (GLMM) with repeated measures was used to compare the abundance of egg rafts collected by leaf substrate, day, and their interaction throughout the 39 day period (Eqn. 1), where *β*_*i*_ represents the random effect of site (block), *L*_*j*_ represents the fixed effect of leaf litter species, *D*_*k*_ represents the fixed effect of day, and *LD*_*jk*_ represents their interaction.

1 $$ {y}_{ijk}=\mu +{\beta}_i+{L}_j+{\varepsilon}_{1(ij)}+{D}_k+L{D}_{jk}+{\varepsilon}_{2(ijk)} $$

Day was the repeated variable, and an autoregressive-1 covariance structure with restricted maximum likelihood (REML) was used for estimation of covariance parameters. Orthogonal contrasts were used to test the linear and quadratic components of the quantitative day variable. Tukey’s mean separation test was used for comparisons of leaf detritus species treatment means. Number of egg rafts was log (x + 0.1)-transformed to meet the assumptions of normally, identically, and independently distributed residuals.

### Effect of leaf detritus species on *Cx. pipiens* emergence and development

*Culex pipiens* larvae were obtained by collecting egg rafts from five sites using standard grass infusion-baited oviposition traps. Egg rafts were individually hatched in petri dishes containing deionized water and first instar larvae of *Cx. pipiens* were distinguished from those of *Culex restuans* Theobald based on the presence or absence of a clear area anterior to the sclerotized egg-breaker which is present in *Cx. restuans* and absent in *Cx. pipiens* [39]. *Culex pipiens* larvae collected from different sites were pooled prior to their random allocation to experimental treatments.

To test for the effect of leaf substrate on intraspecific competition, 18 treatments were established with five replicates per treatment (90 containers). Each treatment included one of three densities of first instar larvae of *Cx. pipiens* (10, 20, or 40 per container) and 360 mL infusion of one of the six leaf detritus species in 400 mL tri-pour beakers. Infusions were prepared by fermenting 80 g of fresh leaves of each plant species in 4 L of tap water for 7 days, a standard infusion age used in comparable studies. Fresh leaves constitute a substantial portion of leaf litter inputs in container habitats and are superior food source for mosquito larvae compared to senescent leaves [29]. The containers were monitored daily and pupae were removed from containers and housed individually in cotton-sealed plastic vials with deionized water. The experiment was conducted under ambient conditions of 25 °C, 70 % relative humidity, and a 16:8 (L:D) photoperiod. Adults were sorted by sex and date of eclosion and their wing lengths determined. Time to eclosion and adult body size are important determinants of the potential for disease transmission (i.e., “vectorial capacity”) in mosquitoes [40–43]. Rapid time to eclosion facilitates high emergence rates of mosquitoes even from ephemeral habitats, while adult body size is directly related to longevity and potential to transmit viruses; only a vector that survives the duration of the viral extrinsic incubation period can infect a human or wildlife host [44].

Separate Multivariate Analysis of Variance (MANOVA) tests were used to test for the fixed effects of leaf detritus species, competition, and their interaction on male and female development time to eclosion and adult wing length. Standardized canonical coefficients (SCCs) were used to describe the relative contributions of development time and wing length to significant treatment effects as well as the relationship between the two variables. A GLMM with a factorial treatment structure was used to test the fixed effects of leaf species, competition, and their interaction on *Cx. pipiens* emergence rates (male and female combined) with Tukey’s separation of means. Development time and wing length were log (x + 0.1)-transformed and emergence data were arcsine-square root (x)-transformed to meet the assumptions of the analysis.

### Relationship between leaf detritus species and microbial composition and abundance

Before adding larvae to treatments as described above, 2 mL aliquots of 7 day old leaf infusions were taken from each container and stored at−80 °C until further processing. Genomic DNA was extracted from the samples using the UltraClean® Soil DNA Isolation Kit (Mo Bio Laboratories Inc., Carlsbad CA, Cat. No. 12800-50) according to the manufacturer’s instructions. Real-time polymerase chain reaction was conducted to detect total bacterial abundance and abundance of seven bacterial phyla (α-proteobacteria, β-proteobacteria, γ-proteobacteria, firmicutes, acidobacteria, actinobacteria, and bacteroidetes) in aliquots of leaf infusions, according to the methods described in [23].

The Shannon diversity index (H) was used to calculate bacterial diversity at the phylum level associated with each leaf detritus species. Further analysis was carried out in three steps. First, GLMs were used to compare total bacterial abundance and bacterial diversity among leaf species, with Tukey’s separation of treatment means. Second, multiple linear regression models were used to assess the relationship between bacterial abundance, bacterial diversity, larval density, and their interaction and mosquito emergence rates, and the relationship between bacterial abundance, bacterial diversity, and their interaction and oviposition rates. Finally, multiple linear regression models were used to identify specific bacterial phyla that are related to mosquito emergence and oviposition rates with phylum-level abundance of seven bacterial phyla as independent effects. Emergence rates were arcsine-square root (x)-transformed and oviposition rates and bacterial abundance and diversity measures were log (x + 0.1)-transformed to meet the assumptions of all tests.

## Results

### Attractiveness of leaf species for *Cx. pipiens* oviposition

**Fig. 1:**
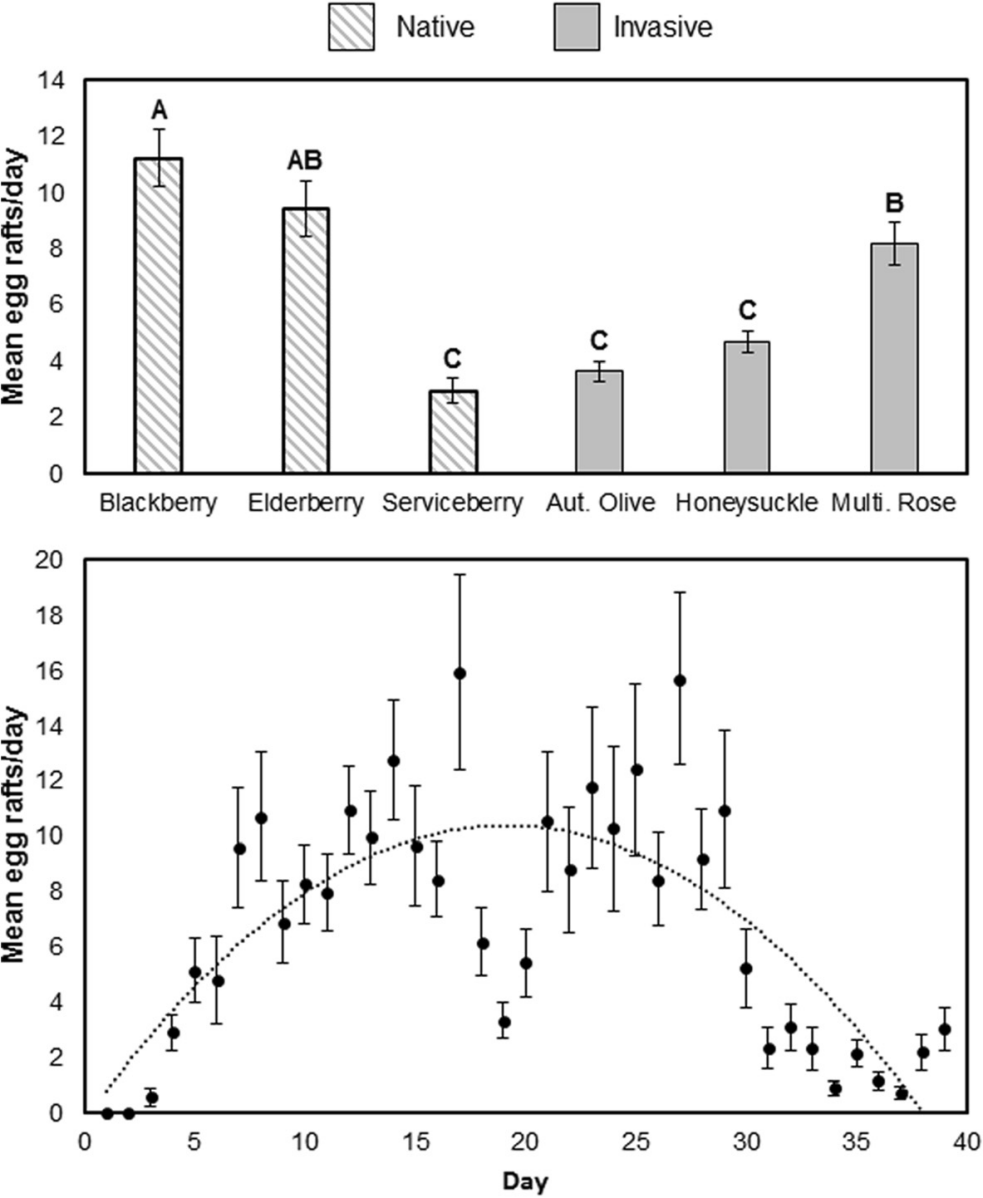
Mean (±1 standard error) for *Culex pipiens* egg rafts collected in oviposition traps per day from June 24 to August 5, 2013 (6 weeks) by leaf detritus treatment. Letters indicate significant pairwise differences at α = 0.05

The number of egg rafts laid in oviposition traps containing the leaves of different native and invasive shrubs varied within and among leaf detritus species over the collection period, with significant effects of leaf species (F = 7.25; df = 5, 20; *P* = 0.0005) and day (F = 23.70; df = 39, 912; *P* < 0.0001) but not their interaction (Fig. 1). Throughout the experiment, the greatest number of egg rafts were collected in blackberry and elderberry leaf infusion, the lowest number of egg rafts per day were collected from water containing serviceberry, autumn olive, and honeysuckle leaves, and an intermediate number of egg rafts were collected from water containing multiflora rose. We found a strong quadratic effect of time (F = 586.89; df = 1, 912; *P* < 0.0001) and a significant though less important linear effect (F = 19.79; df = 1, 912; *P* < 0.0001) of time. The polynomial function relating oviposition response (y) to time (x) is given by

2 $$ {y}_i=-0.029{x}^2+\kern0.5em 1.117x-0.308 $$

Thus, all leaf detritus species initially increased in attractiveness and then declined in attractiveness after the critical point at 19 days.

### Effect of leaf detritus species on *Cx. pipiens* emergence and development

**Fig. 2:**
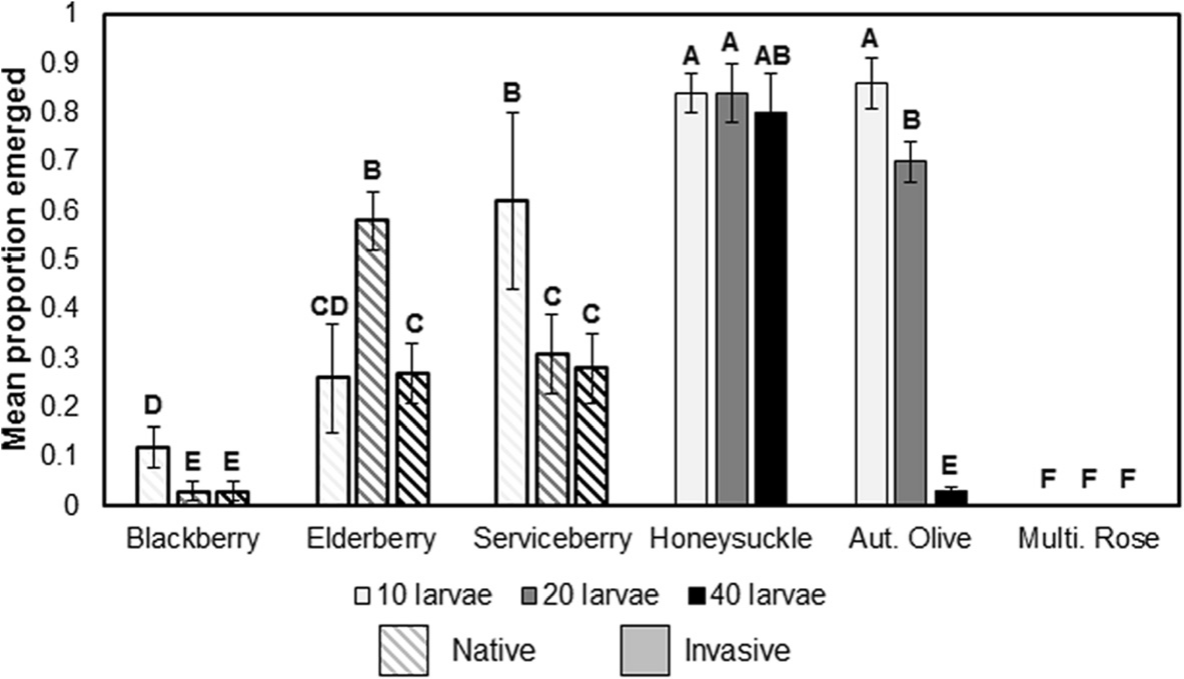
Mean (±1 standard error) for *Culex pipiens* male and female emergence rates across intraspecific competition by leaf detritus treatments. Letters indicate significant pairwise differences at α = 0.05

Mosquito emergence rates varied across leaf detritus types and larval densities, with a significant interaction between leaf species and density (F = 7.33; df = 10, 72; *P* < 0.0001; Fig. 2). The lowest emergence rates were observed in blackberry and multiflora rose infusion; no adults emerged in the latter leaf detritus species at any larval density. The highest emergence rates were observed in honeysuckle and autumn olive infusions, although autumn olive-reared mosquitoes experienced a significant decline in emergence at the highest density while honeysuckle infusion mitigated the deleterious effects of intraspecific competition even at high larval densities. Among all other leaf species except for elderberry, higher larval densities yielded significantly lower emergence rates than lower larval densities.

**Fig. 3:**
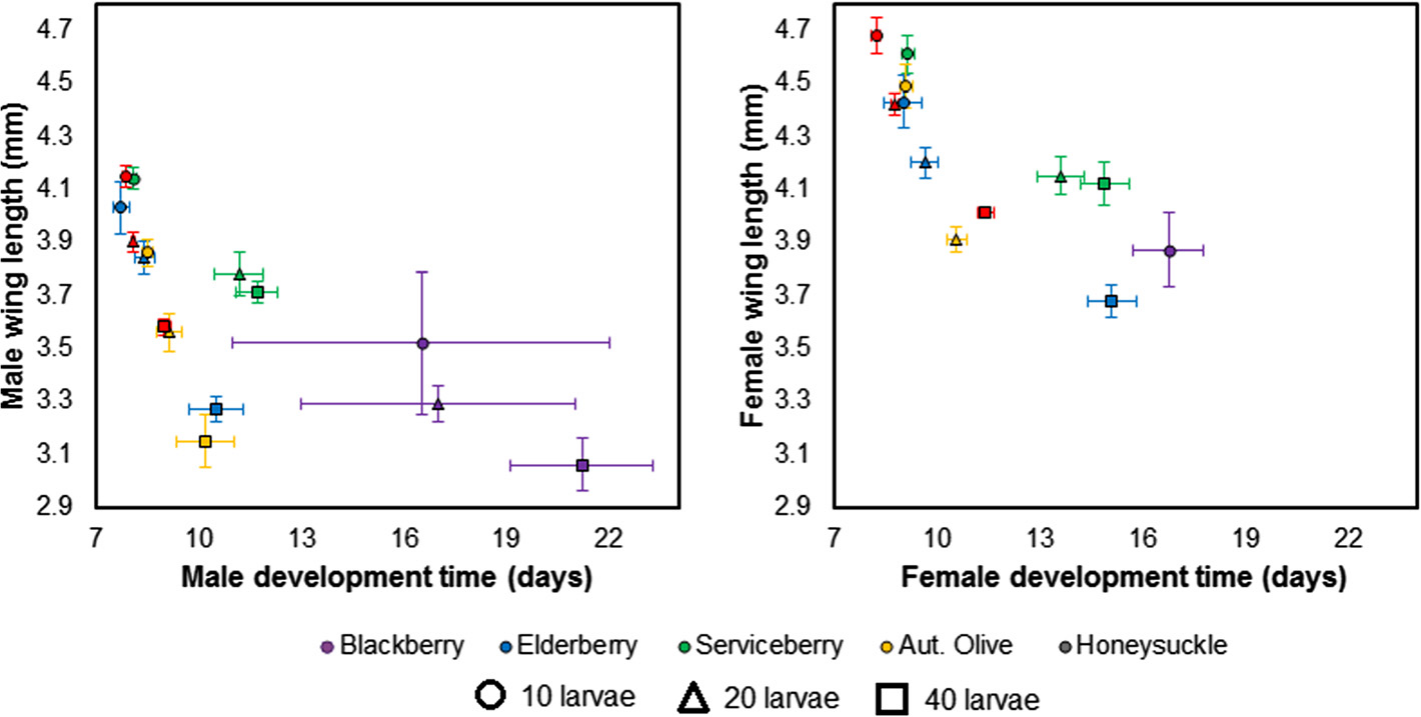
Mean (±1 standard error) for *Culex pipiens* female and male time to eclosion and wing length across intraspecific competition by leaf detritus treatments. The following treatments were excluded because no females survived to eclosion: all multiflora rose treatments; blackberry: 20 larvae and 40 larvae; autumn olive: 40 larvae

Development time to adult eclosion and adult wing length also were influenced by leaf detritus type and larval density, with significant interactions between leaf species and density for females (F = 5.05; df = 16, 644; *P* < 0.0001) and males (F = 3.03; df = 16, 614; *P* < 0.0001; Fig. 3). SCCs show that wing length and development time both contributed strongly to the multivariate effect for females, while wing length explained most variance for males (Table 1). The longest times to eclosion for females were observed in blackberry infusions, while the shortest times to eclosion were observed in honeysuckle infusions. Similarly, the shortest wing lengths for both females and males were observed in blackberry-reared mosquitoes, while the longest wing lengths were observed in honeysuckle-reared mosquitoes. Multiflora rose infusions were excluded from this analysis because no mosquitoes survived in these treatments. Time to eclosion generally increased and adult wing length decreased with higher larval densities, although as in the case for emergence, honeysuckle infusion mitigated the effects of intraspecific competition on development time in particular.

**Table 1:** Multivariate Analysis of Variance (MANOVA) for the effect of leaf detritus species and intraspecific competition on Culex pipiens female and male time to eclosion (days) and wing length (mm). Standardized canonical coefficients (SCCs) describe the relative contribution of each life history trait to the multivariate effect

Sex	Variable	df	Pillai’s trace	P	SCCs	
					Eclosion time	Wing length
Female	Leaf	8	22.07	<0.001*	1.41	−0.33
	Competition	4	18.81	<0.001*	1.01	−0.94
	Leaf*Competition	16	5.05	<0.001*	−0.92	1.02
Male	Leaf	8	26.52	<0.001*	1.25	−0.27
	Competition	4	22.79	<0.001*	−0.44	1.21
	Leaf*Competition	16	3.03	<0.001*	0.38	1.46

Asterisks indicate statistically significant effects at α = 0.05

### Relationship between leaf detritus species and microbial composition and abundance

**Fig. 4:**
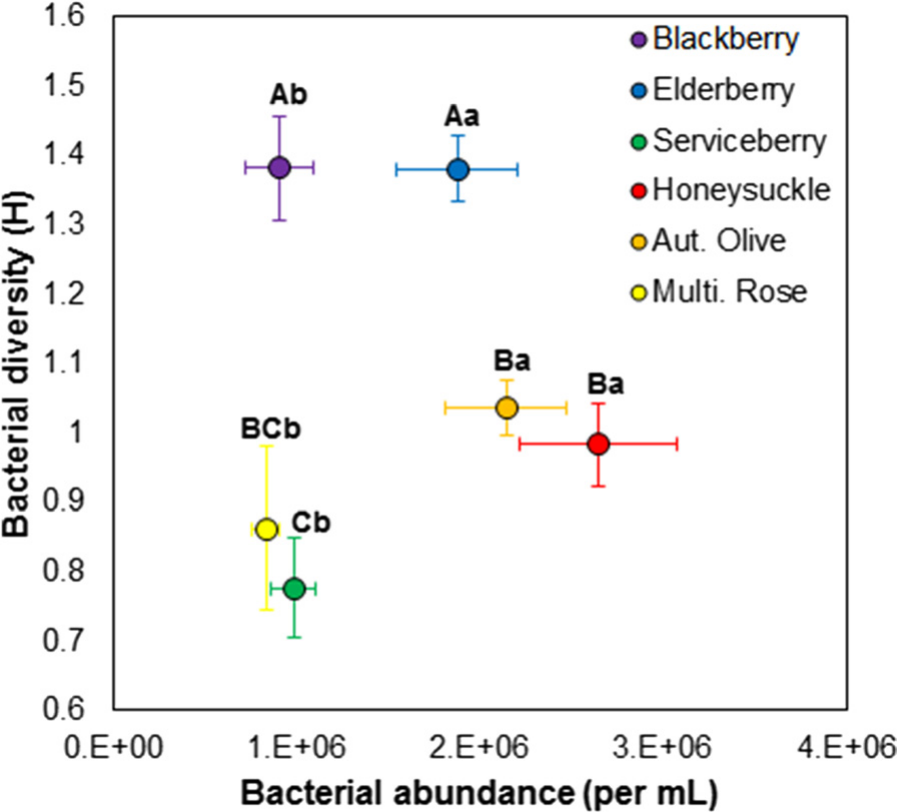
Mean (±1 standard error) for cumulative abundance and Shannon’s diversity index (H) of bacteria by leaf detritus treatment. Upper case letters indicate significant pairwise differences at α = 0.05 for bacterial diversity; lower case letters indicate significant pairwise differences for bacterial abundance

Total abundance (F = 7.24; df = 5, 24; *P* = 0.0003) and diversity of bacterial flora (F = 12.30; df = 5, 24; *P* < 0.0001) varied significantly with leaf detritus species (Fig. 4). A high abundance of bacteria was present in honeysuckle, autumn olive, and elderberry infusion, while a low abundance was present in multiflora rose, blackberry, and serviceberry infusions. High phylum-level diversity of bacteria was present in blackberry and elderberry infusions, while low diversity was present in honeysuckle, multiflora rose, autumn olive, and serviceberry infusions.

Total bacterial abundance and its interaction with bacterial diversity were positively correlated to mosquito emergence rates, with no significant interaction between these effects and larval density (Table 2). Interestingly, the direction of the abundance and diversity interaction was negative, indicating that when bacterial diversity is increased, the positive effect of bacterial abundance on emergence is decreased and vice versa. In particular, abundance of β-proteobacteria was negatively correlated with rate of mosquito emergence while bacteroidetes and acidobacteria were positively correlated with emergence (Table 3). Again, these effects did not interact with larval density.

**Table 2:** Multiple linear regressions for the effect of total bacterial abundance, bacterial diversity, larval density (10, 20, and 40 larvae), and their interaction on Culex pipiens female and male emergence rate and oviposition rates

Variable	Emergence rate		Oviposition rate	
	T	P	T	P
Intercept	−1.22	0.2282	−1.37	0.1877
Abundance	4.78	<0.0001*	−0.75	0.4644
Diversity	0.59	0.5562	2.20	0.0411*
Density	−2.76	0.0073*	-	-
Abundance*Diversity	−2.71	0.0090*	−0.77	0.4534
Abundance*Density	−0.73	0.4706	-	-
Diversity*Density	0.03	0.9794	-	-
Abundance*Diversity*Density	−0.02	0.9836	-	-

Asterisks indicate statistically significant effects at α = 0.05

**Table 3:** Multivariate linear regressions for the effect of phylum-level bacterial composition, larval density (10, 20, and 40 larvae), and their interaction on Culex pipiens female and male emergence and oviposition rates

Variable	Emergence rate		Oviposition rate	
	T	P	T	P
Intercept	3.80	0.0003*	7.18	<0.0001*
α-proteobacteria	−1.69	0.0958	−0.36	0.7258
Bacteroidetes	3.86	0.0003*	−2.10	0.0580
Firmicutes	−1.07	0.2867	0.60	0.5572
β-proteobacteria	−2.75	0.0077*	0.93	0.3726
Acidobacteria	1.98	0.0437*	0.21	0.8338
γ-proteobacteria	0.49	0.6263	−0.39	0.7037
Actinobacteria	−0.22	0.8273	1.43	0.1788
Density	−3.03	0.0035*	-	-
Density*α-proteobacteria	0.08	0.9365	-	-
Density*Bacteroidetes	2.39	0.0196*	-	-
Density*Firmicutes	1.06	0.2944	-	-
Density*β-proteobacteria	−1.44	0.1560	-	-
Density*Acidobacteria	0.97	0.3370	-	-
Density*γ-proteobacteria	0.20	0.8437	-	-
Density*Actinobacteria	−0.67	0.5068	-	-

Asterisks indicate statistically significant effects at α = 0.05

Bacterial diversity but not total bacterial abundance or their interaction was positively correlated to mosquito oviposition rate (Table 2). No particular bacterial phyla were identified that were significantly correlated to oviposition rate (Table 3).

## Discussion

Using a combination of laboratory and field experiments, we identified two invasive shrubs that may promote growth and emergence of an important mosquito vector relative to native shrub species by improving the nutritional quality of the larval environment via leaf detritus inputs. *Culex pipiens* emergence rates were significantly higher in leaf infusions of honeysuckle and autumn olive compared to the other shrub species. We also noted that the deleterious effects of intraspecific larval competition were mitigated in honeysuckle treatments. The reduction of this important constraint on mosquito population carrying capacity [45] indicates that this invasive leaf species may support higher mosquito densities in heavily invaded areas. Similarly, mosquitoes reared in honeysuckle and autumn olive infusion had the shortest development time to eclosion and the longest adult wing lengths in both females and males compared to the other leaf detritus species. These life history characteristics are positively associated with efficiency of mosquito-borne pathogen transmission (i.e., “vectorial capacity”) [40, 43]. Therefore, the widespread distribution of these two invasive shrubs in North America raises concerns regarding their impact on the transmission of several mosquito-borne viruses such as West Nile virus and St. Louis encephalitis.

Although not the most attractive leaf detritus species observed in the experiment, honeysuckle and autumn olive were by far the most favorable leaf detritus species for development and emergence of laboratory-reared mosquitoes. These results complement a growing body of field studies that suggest landscaping with exotic – and potentially invasive – plants has the potential to influence local larval and adult mosquito abundance and distribution [46]. For instance, the presence of ornamental lawn shrubs, including many invasive species, is positively associated with abundance of *Cx. pipiens* and *Cx. restuans* larvae in roadside storm water catch basins [47]. However, it is noteworthy that our experiments also identified a third invasive plant, multiflora rose, that is lethal to *Cx. pipiens*, suggesting that the effect of displacement of native plants by invasive species has neither uniformly positive nor negative impacts on the abundance and distribution of mosquitoes.

Our comparisons of native and invasive leaf detritus species on larval development and oviposition facilitated the discovery of a naturally-occurring ecological trap for *Cx. pipiens*. Ecological traps occur due to a mismatch between the attractiveness of a habitat and its quality for reproduction [48]. The greatest number of egg rafts was collected in water containing leaves of blackberry, a native plant species found throughout the geographic range of *Cx. pipiens*. However, in laboratory assays, exceptionally low mosquito emergence rates were observed in blackberry infusions, with fewer than 20 % of larvae surviving to eclosion even at the lowest larval density. Blackberry-reared mosquitoes also exhibited significantly longer development times to eclosion and the shortest adult wing lengths compared to those exposed to other leaf detritus species. Infusion of multiflora rose, an exotic shrub of limited importance in the Midwest but highly invasive in the northeastern United States [49, 50], similarly yielded lower emergence with no mosquitoes developing to eclosion across all density treatments. However, gravid females were better able to discriminate against this leaf detritus species and consequently water containing multiflora rose leaves collected fewer egg rafts than water containing blackberry leaves. Future research will determine whether exploitation of this ecological trap may yield a novel “attract-kill” approach to control mosquito larvae in closed aquatic environments, such as rain barrels, buckets, and storm water catch basins.

A potential mechanism to explain both asymmetrical oviposition rates and emergence and development rates of mosquitoes with respect to leaf detritus species and differences in the associated microbial flora. Honeysuckle, autumn olive, and elderberry infusions contained greater abundances of bacterial flora than multiflora rose, serviceberry, and blackberry infusion, an observation that likely reflects more rapid leaf decomposition rates among the former species [32, 33], yielding larger amounts of bacteria. For the most part, mosquito emergence reflected this pattern; the notable exception was serviceberry infusion, in which a moderate proportion of mosquitoes developed to eclosion despite low bacterial abundance. This result suggests total bacterial abundance may be a good indicator of habitat quality under most but not all conditions [21]. Microbial abundance also varied significantly between different phyla across leaf detritus species, and was statistically correlated to mosquito emergence. In particular, β-proteobacteria was negatively correlated with emergence rates while bacteroidetes and acidobacteria were positively correlated with emergence rates. This result is consistent with studies of other mosquitoes; for example, *Aedes triseriatus* Say and *Culex tarsalis* Coquillett appear to feed preferentially on bacteroidetes [51, 52].

Further, irrespective of bacterial abundance, phylum-level bacterial diversity was positively correlated with oviposition rates. This result supports previous research which indicates that mosquito oviposition site discovery and selection may be mediated by the odors produced by a higher diversity of bacteria [22, 27]. Thus, we propose that optimal habitats for mosquito production may contain both higher bacterial abundance and higher bacterial diversity, which jointly facilitate discovery of the habitat and the emergence and development of larvae within. Meanwhile, high-diversity and low-abundance habitats such as blackberry-infused water may constitute ecological traps, while low-diversity and high-abundance habitats such as honeysuckle-infused water appear to attract fewer gravid females but promote higher rates of mosquito emergence. It is possible that observed differences in oviposition and emergence rates were not driven by bacterial diversity per se, but rather variation in bacterial community composition between leaf species. It also is noteworthy that oviposition and emergence rates associated with different leaf species may be influenced by phytochemicals unrelated to microbial flora; for example, tannins are abundant in blackberry leaves [53, 54] and are known to be toxic to mosquitoes in other systems [55, 56]. However, these hypotheses were not tested thoroughly in our current experiment.

Our understanding of the mechanistic role potentially played by variation in microbial growth is limited by our focus on a higher taxonomic level of bacteria (i.e., phylum) and at the exclusion of fungi and other microbes that may be consumed by mosquito larvae. Additional studies are required to identify the particular microbial taxa that may be involved in determining mosquito emergence and oviposition site selection. Further, our molecular approach is subject to limitations such as potential underestimation of bacterial abundance due to DNA extraction bias and PCR bias [57, 58]. However, important conclusions can still be made; our findings corroborate previous research which has demonstrated that resource type and availability may influence emergence and oviposition. For example, variation in microbial communities among leaf detritus treatments have been shown to be related to *Culex mollis* Dyar life history traits [59], and binary choice laboratory assays revealed that *Aedes aegypti* Linnaeus oviposition site selection may be related to extracts released by the microbes present in infusions of different leaf species [27].

## Conclusions

In summary, we observed elevated emergence rates and more rapid development among *Cx. pipiens* mosquitoes reared in infusions of honeysuckle and autumn olive leaves, two exotic, invasive shrubs that occur throughout much of the northeastern and midwestern United States. In contrast, we discovered mosquito emergence was significantly reduced among mosquitoes reared in infusions of native blackberry and exotic multiflora rose leaves compared to those exposed to other leaf detritus species. Our results have applications in two areas. First, our findings that some exotic, invasive shrubs are favorable for mosquito production may be relevant to mosquito control and invasive plant management practices in the geographic range of *Cx. pipiens*. Second, our discovery of a previously unknown ecological trap for an important vector of West Nile virus has the potential to lead to novel alternatives to conventional insecticides in mosquito control, exploiting the apparent “attract-kill” properties of this native plant species.
